# Detailed analysis of an enriched deep intronic *ABCA4* variant in Irish Stargardt disease patients

**DOI:** 10.1038/s41598-023-35889-9

**Published:** 2023-06-09

**Authors:** Laura Whelan, Adrian Dockery, Kirk A. J. Stephenson, Julia Zhu, Ella Kopčić, Iris J. M. Post, Mubeen Khan, Zelia Corradi, Niamh Wynne, James J. O’ Byrne, Emma Duignan, Giuliana Silvestri, Susanne Roosing, Frans P. M. Cremers, David J. Keegan, Paul F. Kenna, G. Jane Farrar

**Affiliations:** 1 The School of Genetics and Microbiology, Trinity College Dublin, Dublin 2, Ireland; 2 Next Generation Sequencing Laboratory, Pathology Department, The Mater Misericordiae University Hospital, Dublin 7, Ireland; 3 Department of Ophthalmology, Royal Victoria Eye and Ear Hospital, Dublin 2, Ireland; 4 Mater Clinical Ophthalmic Genetics Unit, The Mater Misericordiae University Hospital, Dublin 7, Ireland; 5 Department of Human Genetics, Radboud University Medical Center, Nijmegen, the Netherlands; 6 Language and Genetics Department, Max Planck Institute for Psycholinguistics, Nijmegen, The Netherlands; 7 International Max Planck Research School for Language Sciences, Max Planck Institute for Psycholinguistics, Nijmegen, The Netherlands; 8 Academic Alliance Genetics, Radboud University Medical Center, Nijmegen, and Maastricht University Medical Center+, Maastricht, The Netherlands; 9 National Centre for Inherited Metabolic Disorders, The Mater Misericordiae University Hospital, Dublin 7, Ireland; 10 School of Medicine, Trinity College Dublin, Dublin 2, Ireland; 11 Centre for Experimental Medicine, Queen’s University Belfast, Belfast, Northern Ireland UK; 12 School of Medicine, University College Dublin, Dublin 4, Ireland; 13 Department of Ophthalmology, The Royal Victoria Hospital, Belfast, Northern Ireland UK

**Keywords:** Genetics, Sequencing

## Abstract

Over 15% of probands in a large cohort of more than 1500 inherited retinal degeneration patients present with a clinical diagnosis of Stargardt disease (STGD1), a recessive form of macular dystrophy caused by biallelic variants in the *ABCA4* gene. Participants were clinically examined and underwent either target capture sequencing of the exons and some pathogenic intronic regions of *ABCA4*, sequencing of the entire *ABCA4* gene or whole genome sequencing. *ABCA4* c.4539 + 2028C > T, p.[= ,Arg1514Leufs*36] is a pathogenic deep intronic variant that results in a retina-specific 345-nucleotide pseudoexon inclusion. Through analysis of the Irish STGD1 cohort, 25 individuals across 18 pedigrees harbour *ABCA4* c.4539 + 2028C > T and another pathogenic variant. This includes, to the best of our knowledge, the only two homozygous patients identified to date. This provides important evidence of variant pathogenicity for this deep intronic variant, highlighting the value of homozygotes for variant interpretation. 15 other heterozygous incidents of this variant in patients have been reported globally, indicating significant enrichment in the Irish population. We provide detailed genetic and clinical characterization of these patients, illustrating that *ABCA4* c.4539 + 2028C > T is a variant of mild to intermediate severity. These results have important implications for unresolved STGD1 patients globally with approximately 10% of the population in some western countries claiming Irish heritage. This study exemplifies that detection and characterization of founder variants is a diagnostic imperative.

## Introduction

Autosomal recessive Stargardt disease (STGD1) is caused by biallelic variants in the ATP-binding cassette transporter type A4 (*ABCA4*) gene^1^ and is characterized by progressive loss of central vision^2^. It is the leading type of inherited macular disease with an approximate incidence of 1-in-10,000^3^. However, this figure is difficult to accurately determine due to the high level of clinical heterogeneity associated with the disease^4^. *ABCA4* (OMIM: #601691) encompasses 50 exons, encoding a protein 2273 amino acids in length. It is expressed in rod, cone, retinal pigment epithelium (RPE) cells and in early endosomal compartments in the RPE^5^. It functions as a flippase for 11-cis and all-trans isomers of N-retinylidene-phosphatidylethanolamine at the rim of rod and cone outer segment disks, allowing for the removal of potentially toxic retinoids^6–8^. If ABCA4 function is impaired, these by-products and other bisretinoids cumulatively termed lipofuscin, accumulate in the RPE, causing dysfunction and cell death followed by impairment and eventually loss of photoreceptors^9–12^.

The vast phenotypic spectrum encompassed by STGD1 is caused by the high level of allelic heterogeneity observed in *ABCA4* and the wide range of effects of variants. The ClinVar database currently lists 651 individual variants with an associated severity rating of likely pathogenic or pathogenic within the *ABCA4* gene^13^. A meta-analysis has listed over 2200 incidents of pathogenic *ABCA4* variants to date which are publicly available at www.lovd.nl/ABCA4^14^. The combination of variants appears to have an impact on the clinical presentation of the patient^15,16^. Disease can range from early onset pan-retinal dystrophy as a result of severe null or null-like variants to onset in adolescence or even later in life due to combinations of severe variants with variants of intermediate severity and milder variants with incomplete penetrance such as *ABCA4* c.5603 T > C, p.Asn1868Ile^17,18^. Genotype–phenotype correlations for pathogenic *ABCA4* variants have yet to be fully elucidated, as have correlations for potential disease modifiers^19^.

As *ABCA4* is a large gene, comprising 128,315 nucleotides, traditional methods of screening have primarily utilized an exon focused sequencing approach. The aim of such studies is to identify disease causing protein coding variants. However, these methods do not ascertain the cause of disease in all cases^20–23^. This highlights the need to broaden the sequencing approach to include non-coding regions of *ABCA4* in order to fully understand the landscape of *ABCA4* related disease^24^. Studies that employ this methodology have identified several novel non-coding pathogenic *ABCA4* variants^25–31^. Variants detected in the non-canonical splice site regions can lead to exon skipping or perturbation of the natural length of the exon^32^. In addition, many non-coding variants are located deep within introns and can result in the incorporation of a pseudoexon into the mature mRNA transcript due to the recruitment of splicing machinery to cryptic splice sites^24,33^. These pseudoexons often disrupt the reading frame or contain premature stop codons, potentially causing the transcript to undergo nonsense mediated decay^34^.

The aberrant splicing patterns identified as a result of intronic variants often do not occur in all transcripts, with a degree of wild-type mature mRNA produced in some incidents^24^. This can depend on the level of similarity of the model utilized for functional analyses to the retina^25,35,36^. Thus, considerable attention should be paid to both the model and the ratio of wild type to mutant transcript observed in order to understand disease manifestation and variant severity. However, it is imperative that follow up clinical studies are carried out to ascertain the true contribution of these variants to disease. Examples include *ABCA4* c.5461-10 T > C which has been classified as severe based functional studies using patient derived fibroblasts^37^ and photoreceptor precursor cells (PPCs) as well as the clinical presentation of four homozygous individuals^38^. Matynia et al. utilised patient induced pluripotent stem cell derived RPE cell models to investigate 4 genetically unresolved STGD1 cases. Subsequently, they identified the cause of disease in 2 of these patients and classified their severity using transcriptomics^39^. Additionally, *ABCA4* c.5196 + 1137G > A was examined in keratinocytes derived from patients heterozygous for this variant resulting in a 73 nucleotide pseudoexon inclusion^33^. It has since been examined in patient-derived PPCs and has been categorized as a variant of intermediate severity following detailed genotype–phenotype analysis in a substantial cohort of patients^36^.

A landmark study on *ABCA4* recently provided the most comprehensive analysis of genetic variation in the entire *ABCA4* gene to date^24^. In this study, seven patients harboured *ABCA4* c.4539 + 2028C > T, p.[= ,Arg1514Leufs*36] of which, five were patients recruited from Ireland. In the current study, through retrospective and continued analyses of the Irish STGD1 cohort, a total of 25 individuals have been identified to date, including two individuals who carry this variant homozygously. This, in conjunction with an evaluation of population controls from Ireland, suggests significant enrichment of this variant in the Target 5000 STGD1 cohort. *ABCA4* c.4539 + 2028C > T was first reported by Braun et al.^33^, with functional analyses carried out by Albert et al.^25^. RNA extracted from patient-derived PPCs was examined with a 345-nucleotide pseudoexon inclusion observed in ~ 15% of all transcripts and ~ 30% of transcripts from this allele. A comprehensive clinical characterization of a group of patients with *ABCA4* c.4539 + 2028C > T is provided. Impact of the variant on disease severity is evaluated through assessment of the phenotype of homozygous patients versus those who carry the variant *in trans* with a null or null-like variant. In addition, we phenotypically compare these individuals to those who carry *ABCA4* variants of known severity *in trans* with a null or null-like variant^15^.

## Results

### ABCA4 c.4539 + 2028C > T is enriched in the Irish STGD1 population

**Figure 1:**
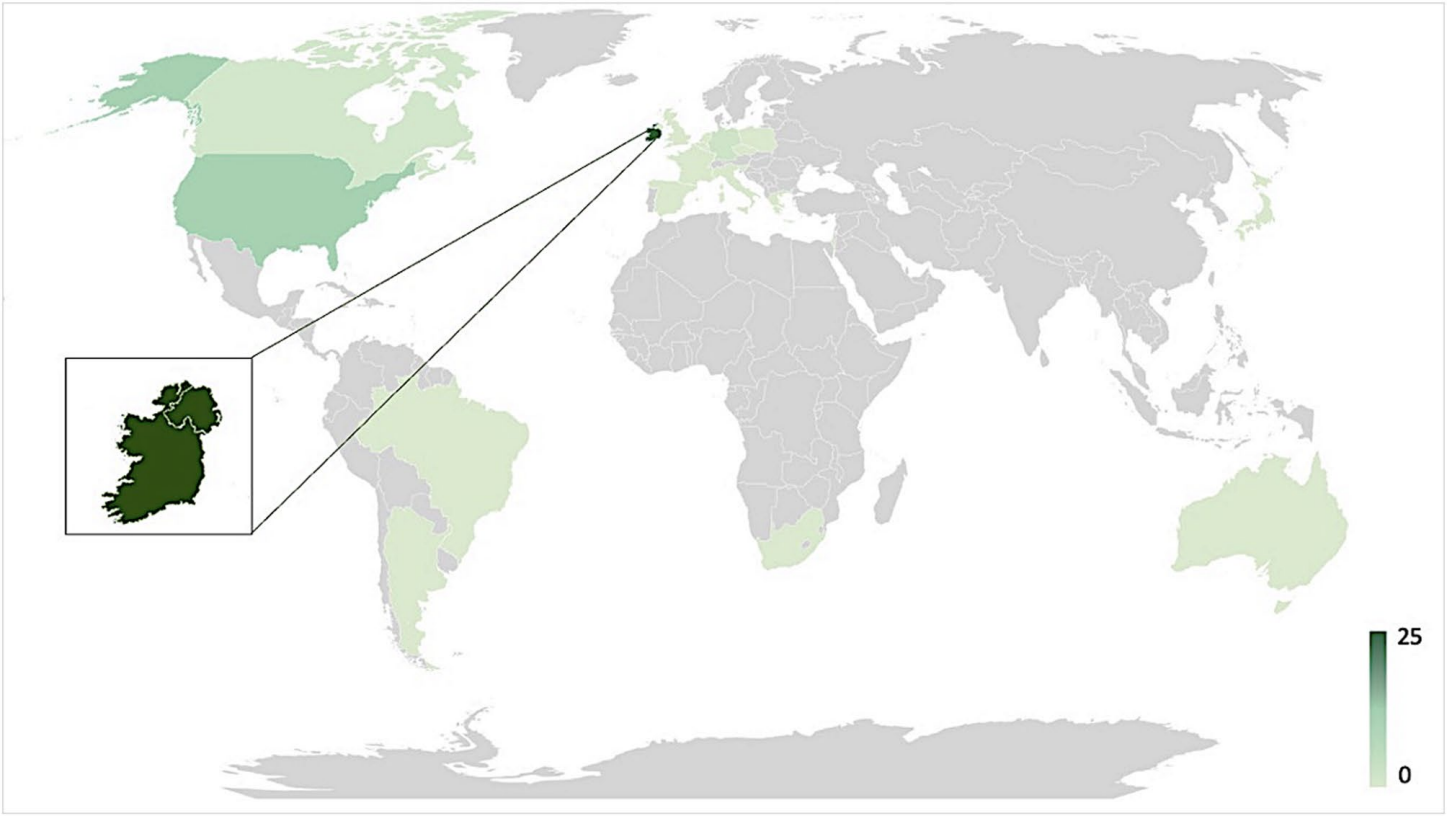
Distribution of *ABCA4* c.4539 + 2028C > T cases worldwide from publications and the Target 5000 (Irish national registry for inherited retinal disease patients) database. The lightest shade of green represents countries where the region harbouring the variant has been sequenced, but no cases were identified. 1 case was detected in Canada, 3 cases were detected in German cohorts, 11 in cohorts in the United States and 25 cases in Ireland including two homozygous individuals^23,29,32,38^. Data has not been published for countries shaded in grey.

27 alleles of *ABCA4* c.4539 + 2028C > T have now been identified in the *ABCA4* sequenced Irish STGD1 population, making this variant the fourth most frequently detected variant in the genetically resolved Irish STGD1 cohort (Table S2). Sex and genotype information for these patients is provided in Table 1. This includes two homozygous individuals, resulting in a total of 25 patients carrying *ABCA4* c.4539 + 2028C > T in Ireland to date. A total of 15 other incidents of the variant have been identified across 5 STGD1 studies in 3,940 alleles tested globally^24,30,33,40,41^ (Fig. 1). *ABCA4* c.4539 + 2028C > T has a population frequency of 0.00003943 in gnomAD (v3.1)^42^, totaling 6/152,182 alleles. A population frequency of 0.000044 was observed in a recent study of 5579 bi-allelic STGD1 patients^43^. Of the 27 alleles of *ABCA4* c.4539 + 2028C > T, 24 of these had the variant *ABCA4* c.302 + 68C > T detected concurrently. *ABCA4* c.302 + 68C > T has an allele frequency of 0.00004598 in gnomAD (v3.1)^42^. The complex allele consisting of these two variants has been previously reported several times^30,33,44^. We could not determine the length of the shared haplotype between individuals in this study as it is limited by the nature of sequencing that has been performed on these patients. However, supplemental Fig. 1 displays variation identified across the *ABCA4* gene in all individuals who have *ABCA4* c.4539 + 2028C > T and *ABCA4* whole gene sequencing. This illustrates that both homozygotes share extremely similar alleles, and it may be possible that this allele is also present in the individuals who carry this variant in a heterozygous manner. Additionally, the numbers of carriers of the complex allele and the variant of interest only in this study were not sufficient to determine if the presence of the second variant had an additional effect on the severity of the phenotype. In addition, *ABCA4* c.302 + 68C > T does not have a qualitative effect on the resulting mRNA transcript in PPCs^25^. Furthermore, to explore the frequency of the *ABCA4* c.4539 + 2028C > T and *ABCA4* c.302 + 68C > T allele in the Irish population an unaffected control cohort previously subjected to whole genome sequencing has been interrogated, and a single allele harbouring both variants has been observed in the 408 healthy individuals evaluated to date*.* Of note, the two individuals that are homozygous for the *ABCA4* c.4539 + 2028C > T variant are the only homozygotes observed to date globally. Homozygotes for this or any *ABCA4* variant, in principle provide an opportunity to explore the functional consequences of variants in patients.

**Table 1:** Patient ID, Pedigree, sex and genotype information of all individuals harbouring *ABCA4* c.4539 + 2028C > T, p.[= ,Arg1514Leufs*36].

PID	Pedigree	Sex	Allele 1		Allele 2	
			cDNA	Protein	cDNA	Protein
Pt-1^‡^	1	M	c.4539 + 2028C > T	p.[= ,Arg1514Leufs*36]	c.2160 + 1G > C	p.(?)
Pt-2	2	F	c.4539 + 2028C > T	p.[= ,Arg1514Leufs*36]	c.[4222 T > C;4918C > T]	p.[Trp1408Arg;Arg1640Trp]
Pt-3	2	M	c.4539 + 2028C > T	p.[= ,Arg1514Leufs*36]	c.[4222 T > C;4918C > T]	p.[Trp1408Arg;Arg1640Trp]
Pt-4	2	M	c.4539 + 2028C > T	p.[= ,Arg1514Leufs*36]	c.[4222 T > C;4918C > T]	p.[Trp1408Arg;Arg1640Trp]
Pt-5	3	M	c.4539 + 2028C > T	p.[= ,Arg1514Leufs*36]	c.3322C > T	p.(Arg1108Cys)
Pt-6	4	F	c.4539 + 2028C > T	p.[= ,Arg1514Leufs*36]	c.6472A > G	p.(Lys2158Glu)
Pt-7	5	M	c.4539 + 2028C > T	p.[= ,Arg1514Leufs*36]	c.2453G > A	p.(Gly818Glu)
Pt-8	6	M	c.4539 + 2028C > T	p.[= ,Arg1514Leufs*36]	c.3322C > T	p.(Arg1108Cys)
Pt-9^‡^	7	F	c.4539 + 2028C > T	p.[= ,Arg1514Leufs*36]	c.3754G > T	p.(Glu1252*)
Pt-10^†^	8	M	c.4539 + 2028C > T	p.[= ,Arg1514Leufs*36]	c.4539 + 2028C > T	p.[= ,Arg1514Leufs*36]
Pt-11	9	F	c.4539 + 2028C > T	p.[= ,Arg1514Leufs*36]	c.4139C > T	p.(Pro1380Leu)
Pt-12^‡^	10	M	c.4539 + 2028C > T	p.[= ,Arg1514Leufs*36]	c.6449G > A	p.(Cys2150Tyr)
Pt-13	11	F	c.4539 + 2028C > T	p.[= ,Arg1514Leufs*36]	c.161G > A	p.(Cys54Tyr)
Pt-14	12	M	c.4539 + 2028C > T	p.[= ,Arg1514Leufs*36]	c.[4222 T > C;4918C > T]	p.[Trp1408Arg;Arg1640Trp]
Pt-15	12	F	c.4539 + 2028C > T	p.[= ,Arg1514Leufs*36]	c.[4222 T > C;4918C > T]	p.[Trp1408Arg;Arg1640Trp]
Pt-16	12	F	c.4539 + 2028C > T	p.[= ,Arg1514Leufs*36]	c.[4222 T > C;4918C > T]	p.[Trp1408Arg;Arg1640Trp]
Pt-17^‡^	13	F	c.4539 + 2028C > T	p.[= ,Arg1514Leufs*36]	c.3212C > A	p.(Ser1071*)
Pt-18	14	F	c.4539 + 2028C > T	p.[= ,Arg1514Leufs*36]	c.4139C > T	p.(Pro1380Leu)
Pt-19	15	M	c.4539 + 2028C > T	p.[= ,Arg1514Leufs*36]	c.223 T > G	p.(Cys75Gly)
Pt-20	15	M	c.4539 + 2028C > T	p.[= ,Arg1514Leufs*36]	c.223 T > G	p.(Cys75Gly)
Pt-21	16	M	c.4539 + 2028C > T	p.[= ,Arg1514Leufs*36]	c.4139C > T	p.(Pro1380Leu)
Pt-22	17	F	c.4539 + 2028C > T	p.[= ,Arg1514Leufs*36]	c.6743 T > C	p.(Phe2248Ser)
Pt-23^†^	18	F	c.4539 + 2028C > T	p.[= ,Arg1514Leufs*36]	c.4539 + 2028C > T	p.[= ,Arg1514Leufs*36]
Pt-24	18	F	c.4539 + 2028C > T	p.[= ,Arg1514Leufs*36]	c.[4222 T > C;4918C > T]	p.[Trp1408Arg;Arg1640Trp]
Pt-25	18	F	c.4539 + 2028C > T	p.[= ,Arg1514Leufs*36]	c.[4222 T > C;4918C > T]	p.[Trp1408Arg;Arg1640Trp]

The relevant accession numbers for *ABCA4* are NM_000350.2 and NC_000001.11.

*M* male, *F* female.

^†^Homozygous individuals.

^‡^Those who carry a null or null-like variant.

### Clinical characterisation of individuals carrying ABCA4 c.4539 + 2028C > T

Detailed phenotypic data including visual acuity, fundus autofluorescence (FAF), optical coherence tomography (OCT), electroretinography (ERG) and age at onset was collected from all 25 patients harbouring *ABCA4* c.4539 + 2028C > T, where available, including two homozygous individuals. The median age at last examination was 38 (range = 13–70) years. In order to determine the severity of *ABCA4* c.4539 + 2028C > T, two indivduals who carry this variant in a homozygous manner were compared to four individuals who carry this variant *in trans* with a null or null-like variant. The phenotype of these four individuals allows surveyance of the effect of *ABCA4* c.4539 + 2028C > T without residual function from the other allele, while the clinical presentation of the homozygous individuals highlights the direct phenotypic outcome of *ABCA4* c.4539 + 2028C > T without the presence of any additional pathogenic *ABCA4* variants. The individuals who carry this variant *in trans* with a null or null-like variant were also compared to individuals who carry variants of known severity *in trans* with a null or null-like variant as described by Fakin et al.^15^. Details of these variants are included in the methods section. These comparisons were performed in order to gauge the pathogenicity of *ABCA4* c.4539 + 2028C > T on the STGD1 severity spectrum.

#### Visual acuity

The visual acuity of all individuals harbouring *ABCA4* c.4539 + 2028C > T, where available, can be found in Table 2. The median visual acuity of the homozygotes was 6/33.5 at a median age of 47.5, with the female having visual acuity of 6/48 at age 58 and the male having a visual acuity of 6/19 at age 37. By comparison, the median visual acuity of those who harboured *ABCA4* c.4539 + 2028C > T *in trans* with a null or null-like variant was 6/48 (range = 6/36–60, n = 4) at a median age of 36.5 (range = 16–41, n = 4). In addition, the median visual acuity of those who carried mild variants *in trans* with a null or null-like variant was 6/60 (range = 6/7.5–60, n = 9) at a median age of 33 (range = 17–50, n = 9). The median visual of those who carried intermediate variants *in trans* with a null or null-like variant was 6/60 (range = 6/48–75. n = 4) at a median age of 22.5 (range = 13–54, n = 4).

**Table 2:** Pedigree numbers and visual acuity of all individuals harbouring *ABCA4* c.4539 + 2028C > T.

Patient ID	Pedigree	VA–OD	VA–OS
Pt-1^‡^	1	6/36	6/36
Pt-2	2	6/90	6/75
Pt-3	2	6/48	6/48
Pt-4	2	6/120	6/600
Pt-5	3	6/6	6/4.5
Pt-6	4	6/36	6/36
Pt-7	5	6/36	6/120
Pt-8	6	6/90	6/75
Pt-9^‡^	7	6/60	6/36
Pt-10^†^	8	6/19	6/19
Pt-11	9	6/48	6/48
Pt-12^‡^	10	6/60	6/60
Pt-13	11	CF	6/60
Pt-14	12	6/48	6/30
Pt-15	12	6/48	6/48
Pt-16	12	6/48	6/48
Pt-17^‡^	13	6/60	6/60
Pt-18	14	6/60	6/60
Pt-19	15	6/60	6/48
Pt-20	15	6/60	6/60
Pt-21	16	6/38	6/38
Pt-22	17	///	///
Pt-23^†^	18	6/60	6/48
Pt-24	18	6/38	6/38
Pt-25	18	6/7.5	6/7.5

*CF* counting fingers, *VA* visual acuity, *OD* right eye, *OS* left eye, /// not available.

^†^Homozygous individuals.

^‡^Those who carry a null or null-like variant.

#### Fundus autofluorescence and optical coherence tomography

**Figure 2:**
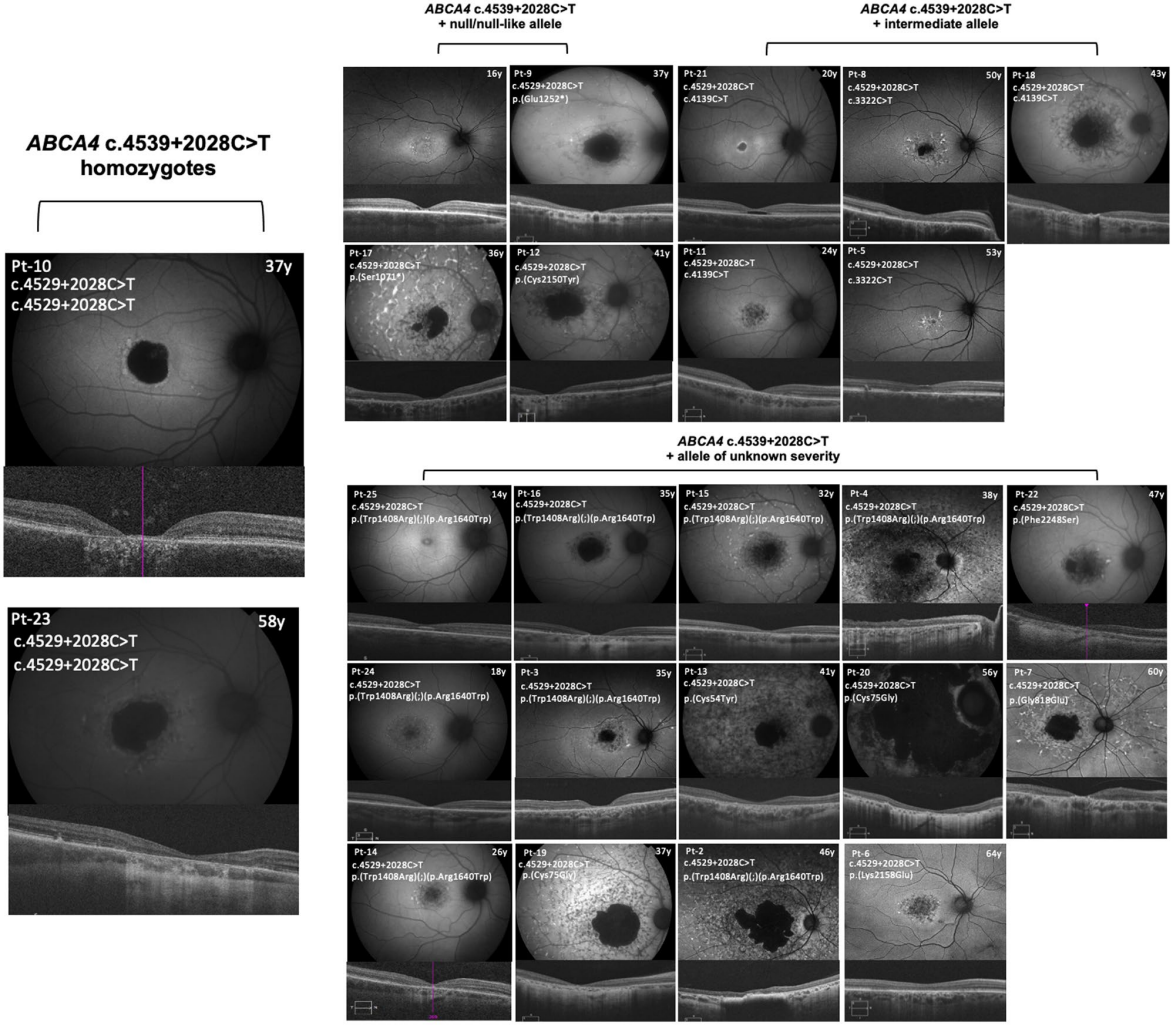
Fundus autofluorescence (FAF) and optical coherence tomography (OCT) analysis of all individuals harbouring *ABCA4* c.4539 + 2028C > T. FAF and OCT is shown for the right eye of each patient, with variant details and age at time of image capture overlaid. c.4539 + 2028C > T homozygotes show focal, well-circumscribed macular hypoautofluorescence surrounded by minimal retinal flecks (mean horizonal width of atrophy on OCT 2596 µm). c.4539 + 2028C > T with an *ABCA4* allele of intermediate severity manifested retinal changes confined mainly to the perifoveal area, whereas c.4539 + 2028C > T in conjunction with a null/null-like allele showed more widespread disease, extending beyond the arcades in older probands. c.4539 + 2028C > T with alleles of unknown pathogenic potential show a range of distribution of retinal pathology, however, the majority have central profound hypoautofluorescence as seen in the c.4539 + 2028C > T homozygotes.

All individuals exhibited fundus autofluorescence (FAF) abnormalities (Fig. 2). In 13/25 individuals, this abnormality extended beyond the vascular arcades. 11/25 individuals were designated a group 1 Fishmann classification^2^, including both homozygous individuals. 9/25 individuals were classified as group 2, four individuals were classified as group 3 and one individual was classified as group 4. A distinctive well-circumscribed circular area of profound atrophy on auto-fluorescent imaging was observed in the majority of the 25 individuals harbouring *ABCA4* c.4539 + 2028C > T, including both homozygous individuals. 20/25 had peripapillary sparing. Peripheral retinal yellow flecks were noted in 9/25 individuals, while central flecks were seen in 23/25 individuals. Of those without flecks, one case (EEJ13, age 56, Fig. 2) had advanced, diffuse retinal atrophy without flecks and the second (EECC41, age 14, Fig. 2) had early foveal RPE disturbance only. In addition, 20/25 individuals had a beaten-bronze appearance and 19/25 exhibited a bull’s eye maculopathy pattern. Symmetry was noted in 21/25 individuals. Details of FAF analysis are provided in Table 3.

**Table 3:** Detailed fundus examination of all individuals harbouring *ABCA4* c.4539 + 2028C > T.

Patient ID	Pedigree	FAF WRT vascular arcades	Bull’s eye pattern	Beaten bronze appearance	Yellow flecks centrally	Flecks peripherally	Peripapillary sparing
Pt-1^‡^	1	Within	Y	Y	Y	N	Y
Pt-2	2	Beyond	N	Y	Y	Y	Y
Pt-3	2	Beyond	Y	Y	Y	N	Y
Pt-4	2	Beyond	N	N	Y	Y	N
Pt-5	3	Within	Y	Y	Y	N	Y
Pt-6	4	Within	Y	Y	Y	N	Y
Pt-7	5	Beyond	N	Y	Y	N	Y
Pt-8	6	Within	Y	Y	Y	N	Y
Pt-9^‡^	7	Beyond	N	Y	Y	Y	Y
Pt-10^†^	8	Within	Y	N	Y	N	Y
Pt-11	9	Within	Y	Y	Y	N	Y
Pt-12^‡^	10	Beyond	Y	Y	Y	Y	Y
Pt-13	11	Beyond	Y	Y	Y	Y	N
Pt-14	12	Beyond	Y	Y	Y	N	Y
Pt-15	12	Within	Y	Y	Y	Y	N
Pt-16	12	Beyond	Y	Y	Y	N	Y
Pt-17^‡^	13	Beyond	Y	Y	Y	Y	N
Pt-18	14	Beyond	Y	Y	Y	N	Y
Pt-19	15	Beyond	N	N	Y	Y	Y
Pt-20	15	Beyond	N	N	N	Y	N
Pt-21	16	Within	Y	Y	Y	N	Y
Pt-22	17	Within	Y	Y	Y	N	Y
Pt-23^†^	18	Within	Y	Y	Y	N	Y
Pt-24	18	Within	Y	N	N	N	Y
Pt-25	18	Within	Y	Y	Y	N	Y

^†^Homozygous individuals.

^‡^Those who carry a null or null-like variant.

*WRT* with respect to, *Y* yes, *N* no.

The foveal photoreceptors were affected on optical coherence tomography (OCT) in all individuals, with none exhibiting foveal sparing (Fig. 2). One participant displayed only mild foveal deterioration, however this individual was 13 years old at the time of multimodal imaging and the severe manifestations of the phenotype may not yet be fully apparent at this age (Fig. 2, Pt-25). It is of note that 16/25 patients exhibited profound foveal atrophy (including both homozygotes), 8/25 presented with a intermediate macular phenotype and 1/25 (Pt-25) with a mild macular phenotype when OCT and FAF phenotype were surveyed. In contrast, 1/25 (Pt-20) 6/25, 3/25 and 14/25 (including both homozygotes) exhibited severe, intermediate, mild and normal peripheral phenotypes respectively. The median central retinal thickness (CRT) of all individuals harbouring *ABCA4* c.4539 + 2028C > T was 121 microns (range = 73–256, n = 25). The median horizontal OCT atrophy area was 3123 microns (range = 860–6000, n = 25). Data for each individual can be found in Table 4.

**Table 4:** Examination of central retinal thickness (CRT) and optical coherence tomography (OCT) atrophy area in all individuals harbouring *ABCA4* c.4539 + 2028C > T.

Patient ID	Pedigree	CRT – OD (microns)	CRT – OS (microns)	OCT atrophy horizontal width OD (microns)	OCT atrophy horizontal width OS (microns)
Pt-1^‡^	1	162	164	1732	1064
Pt-2	2	140	90	6000	6000
Pt-3	2	109	///	2824	3467
Pt-4	2	176	87	6000	6000
Pt-5	3	196	210	2322	2615
Pt-6	4	94	98	3604	3257
Pt-7	5	100	117	6000	5446
Pt-8	6	117	100	2990	3735
Pt-9 ^‡^	7	120	123	4608	4473
Pt-10 ^†^	8	73	66	2189	2182
Pt-11	9	121	119	2234	2162
Pt-12^‡^	10	99	121	4996	4919
Pt-13	11	109	106	6000	6000
Pt-14	12	168	179	2436	2623
Pt-15	12	104	111	3360	2748
Pt-16	12	106	118	3739	3585
Pt-17^‡^	13	68	72	4630	3344
Pt-18	14	65	91	5099	4965
Pt-19	15	184	74	4536	3828
Pt-20	15	99	106	6000	6000
Pt-21	16	189	182	1055	1157
Pt-22	17	197	249	4290	4999
Pt-23^†^	18	225	256	2621	3392
Pt-24	18	115	121	2072	2805
Pt-25	18	200	198	860	1106

6000 microns was the maximum horizontal width reading measurable by OCT and implies a larger field of outer retinal atrophy.

^†^Homozygous individuals.

^‡^Those who carry a null or null-like variant.

///: not available.

**Figure 3:**
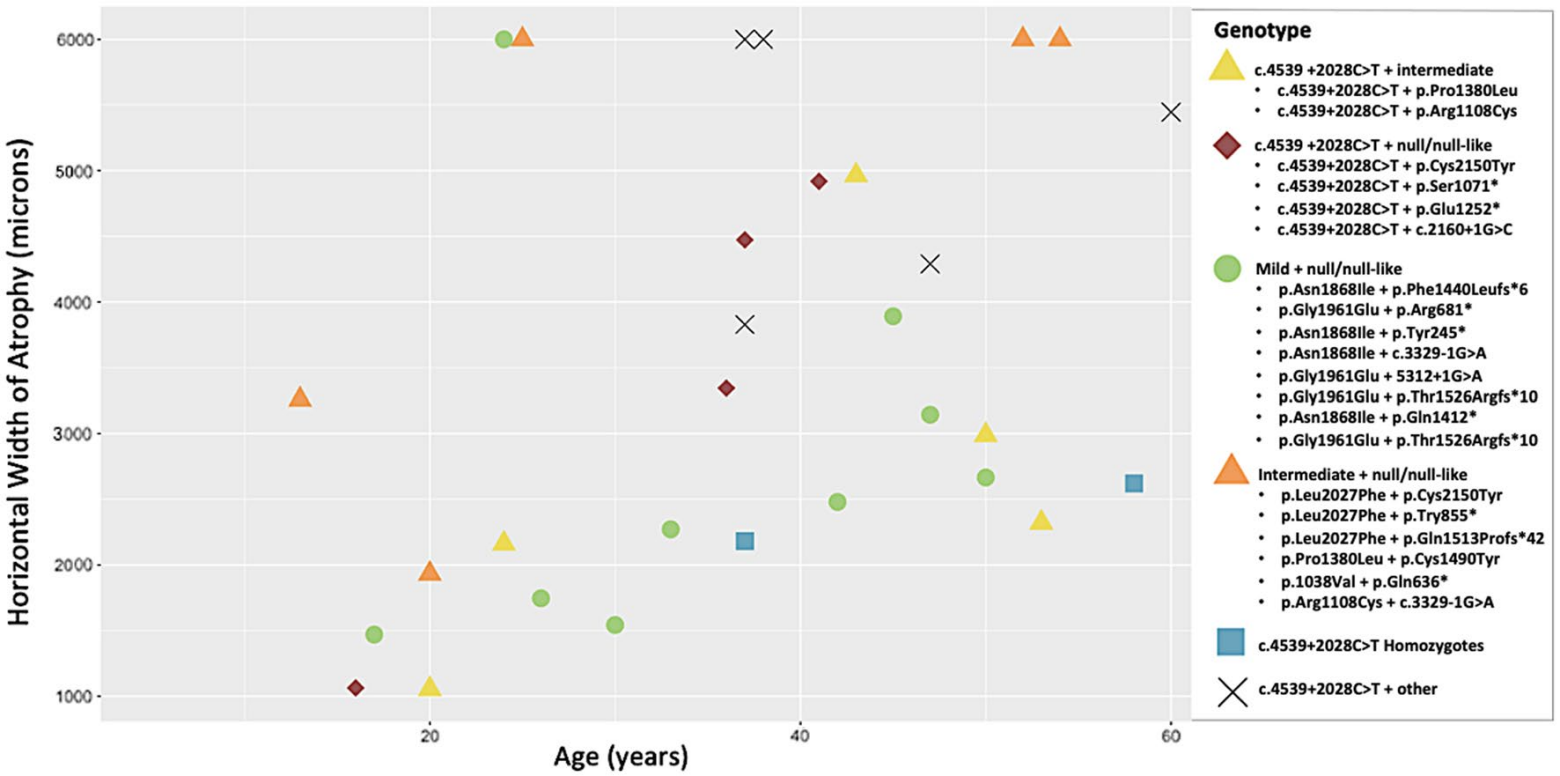
Scatterplot of atrophy area horizontal width vs. age (in years) grouped by genotype. Those who carry *ABCA4* c.4539 + 2028C > T *in trans* with a null or null-like variant (red diamonds) appear to have a larger atrophy area at an earlier or similar age to c.4539 + 2028C > T homozygotes (blue squares). Individuals who carry mild variants *in trans* with a null or null-like variant (green circles) display a smaller atrophy area when compared to those of similar age who carry c.4539 + 2028C > T *in trans* with a null or null-like variant while the atrophy area of those who carry intermediate variants *in trans* with a null or null-like variant (orange triangles) is larger. Other = variants of unknown severity. 6000 microns was the maximum possible measurement via the OCT platform used and suggests a larger field of outer retinal atrophy.

The median CRT, OCT atrophy area, age and sample size for the c.4539 + 2028C > T homozygotes, patients who carried *ABCA4* c.4539 + 2028C > T *in trans* with a null or null-like variant, patients who carried mild variants *in trans* with a null or null-like variant and patients who carried intermediate *in trans* with a null or null-like variant can be found in Table 5. The homozygous individuals have a less severely affected median CRT and OCT atrophy area at a later median age than all three other groups. The patients who carried *ABCA4* c.4539 + 2028C > T *in trans* with a null or null-like variant had median CRT and OCT atrophy areas intermediate relative to that of patients who carried the mild and intermediate variants *in trans* with a null or null-like variant (Fig. 3).

**Table 5:** Median central retinal thickness (CRT), optical coherence tomography (OCT) atrophy area (AA) and age for patients homozygous for *ABCA4* c.4539 + 2028C > T and who carry *ABCA4* c.4539 + 2028C > T *in trans* with a null or null-like variant, mild variants *in trans* with a null or null-like variant and intermediate *in trans* with a null or null-like variant.

Genotype	Median CRT (microns)	Median OCT AA horizontal width (microns)	Median age (years)	Sample size
c.4539 + 2028C > T Homozygotes	164.5 (R:73–256)	2401.6 (R:2182–2621)	47.5 (R:37–58)	2
c.4539 + 2028C > T + null/null-like	122 (R:72–164)	3908 (R:1064–4919)	36.5 (R:21–60)	4
Mild + null/null-like	124 (R:87–195)	2480 (R:1470–6000)	33 (R:13–54)	5
Intermediate + null/null-like	116(R:83–127)	5444 (R:1933–6000)	38.5 (R:17–50)	9

*R* range.

#### Electroretinography

**Figure 4:**
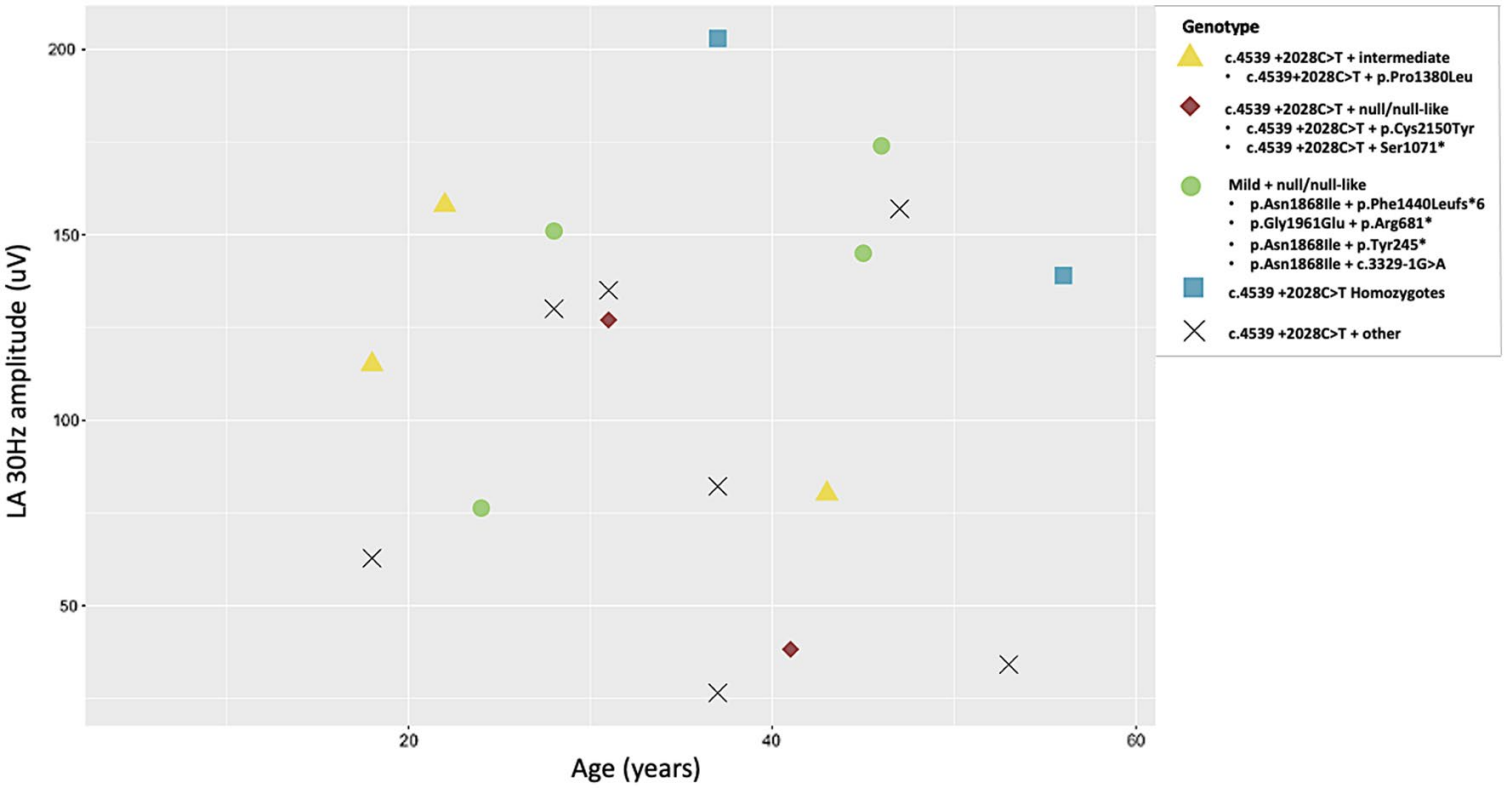
Scatterplot of light adapted (LA) 30 Hz amplitude (uV) vs. age (years) grouped by genotype. Those who carry *ABCA4* c.4539 + 2028C > T *in trans* with a null or null-like variant (red diamonds) have a more attenuated response at an earlier or similar age to c.4539 + 2028C > T homozygotes (blue squares). Individuals who carry mild variants *in trans* with a null or null-like variant (green circles) display a higher reading when compared to those who carry c.4539 + 2028C > T *in trans* with a null or null-like variant of similar or earlier age. Individuals who carry c.4539 + 2028C > T *in trans* with a variant of intermediate severity (yellow triangles) display more marked attenuation of LA 30 Hz amplitude than the homozygotes at earlier ages. However, the sample size is limited. Other = variants of unknown severity. ERG light adapted 30 Hz amplitude lower limit of normal is 125uV.

**Figure 5:**
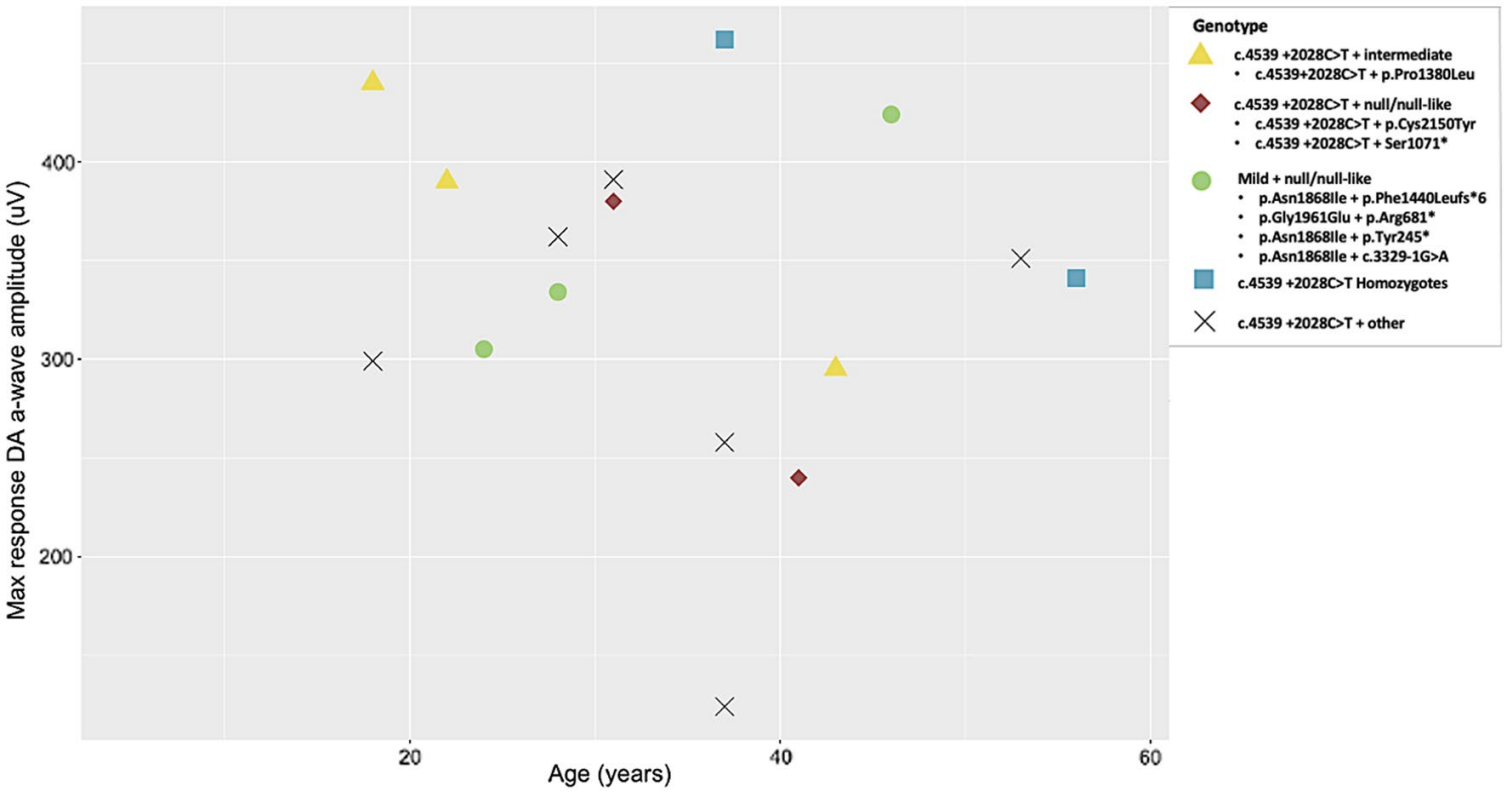
Scatterplot of maximum response dark adapted (DA) a-wave amplitude (uV) vs. age (years) grouped by genotype. Those who carry *ABCA4* c.4539 + 2028C > T *in trans* with a null or null-like variant (red diamonds) retain less function at an earlier or similar age to c.4539 + 2028C > T homozygotes (blue squares). Individuals who carry c.4539 + 2028C > T *in trans* with a variant of intermediate severity (yellow triangle) have a lower reading at earlier ages than the two homozygotes. However, the sample sizes are limited. Other = variants of unknown severity. ERG dark adapted 3 a wave amplitude lower limit of normal is 292 uV.

Electroretinography (ERG) data was available for 14/25 participants (Table 6), all recruited from one institution and examined by the same clinician. The median age at ERG recording was 37 (range = 18–56). In accordance with a published ERG classification system^45^, 9/25 individuals were designated a group 1 status (normal full-field ERG [ffERG]; median age of 31 (range 18–56), including both homozygotes. 4/25 individuals were classified into group 2 (reduced photopic function; median age of 41 (range 18–43). One individual was classified as group 3 (reduced photopic and scotopic function; age 37). ERG results plotted against age can be found in Figs. 4 and 5.

**Table 6:** Electroretinography (ERG) data collected from a subset of individuals harbouring *ABCA4* c.4539 + 2028C > T.

Patient ID	Pedigree	Age at ERG	ffERG group	PERG	ERG DA 3 a wave OD (uV)	ERG DA 3 a wave OS (uV) ERG	ERG LA 30 Hz OD (uV)	ERG LA 30 Hz OS (uV)
Pt-10^†^	8	37	1	N	433	462	186	203
Pt-11	9	22	1	///	348	390	134	158
Pt-12^‡^	10	41	2	///	240	173	38.2	31.8
Pt-13	11	37	3	A	124	111	26.4	25.2
Pt-15	12	28	1	A	330	362	121	130
Pt-16	12	31	3	///	336	392	135	119
Pt-17^‡^	13	31	1	///	380	337	101	127
Pt-18	14	43	2	A	295	269	77.8	80.2
Pt-19	15	37	1	///	178	258	27.6	82.2
Pt-20	15	53	1	///	230	351	34.1	30.6
Pt-21	16	18	1	A	405	440	115	78.8
Pt-22	17	47	2	///	210	267	116	157
Pt-23^†^	18	56	1	A	319	341	128	139
Pt-24	18	18	2	///	241	299	57.2	62.8

ERG dark adapted 3 a wave amplitude lower limit of normal is 292 uV, ERG light adapted 30 Hz amplitude lower limit of normal is 125uV.

^†^Homozygous individuals.

^‡^Those who carry a null or null-like variant.

*ffERG* full field electroretinography, *PERG* pattern electroretinography, *N* normal, *A* abnormal, /// not available, *DA* dark adapted, *LA* light adapted, *OD* right eye, *OS* left eye.

#### Age at onset

**Figure 6:**
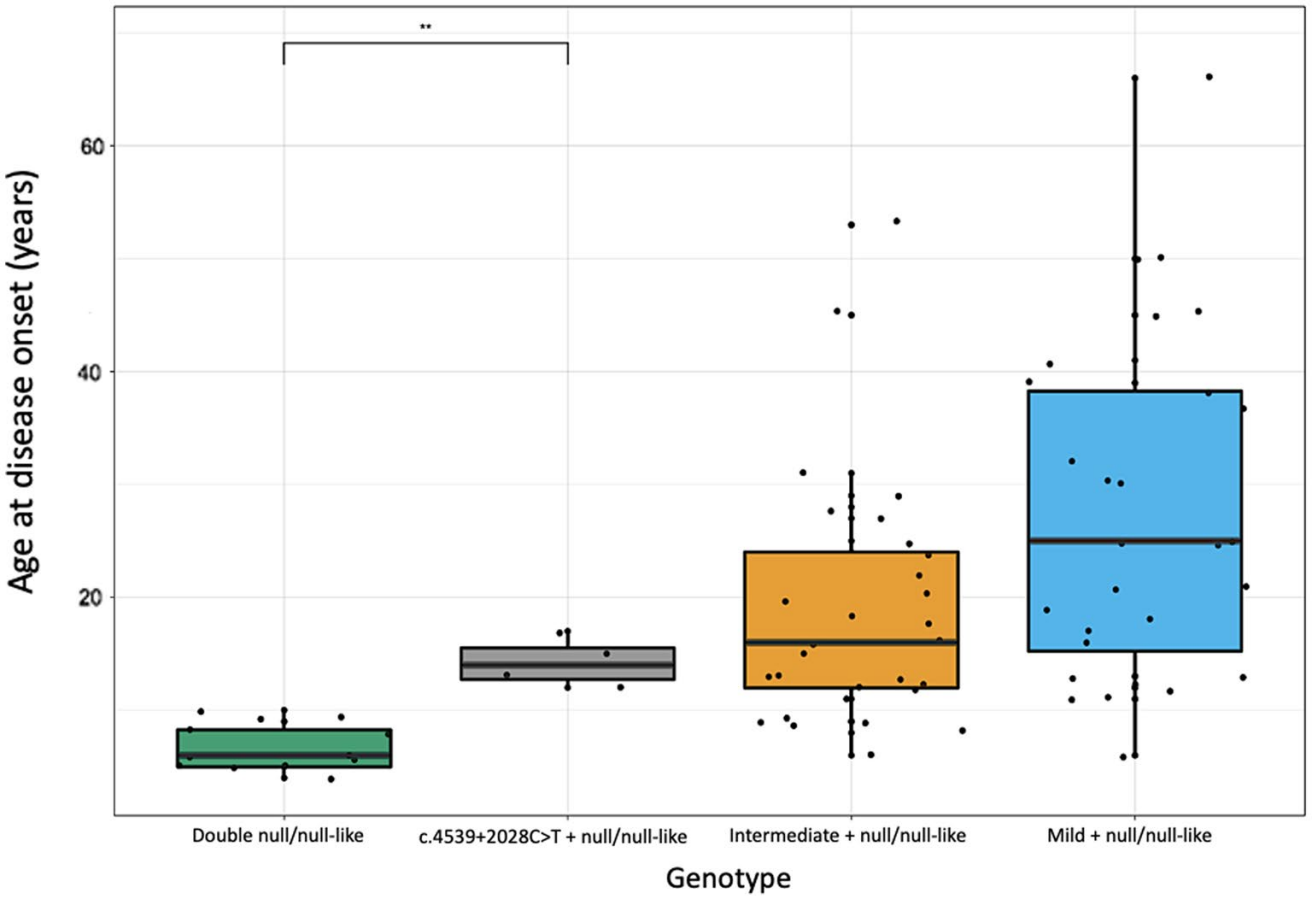
Boxplot illustrating age at disease onset (years) vs. genotype. Patients who carry *ABCA4* c.4539 + 2028C > T *in trans* with a null or null-like variant have a significantly later age at disease onset than double null/null-like individuals (p = 0.0082). The median age at onset of those who carry c.4539 + 2028C > T *in trans* with a null or null-like variant appears to be earlier than that of the patients who carry mild and intermediate variants *in trans* with a null or null-like variant, although sample size is limited.

The median age at onset for those with *ABCA4* c.4539 + 2028C > T was 15 (range = 6–49 years). As age at onset data on *ABCA4* variants of known severity was available through prior publications, these data were used in statistical analyses^15^. In order to assess the severity of *ABCA4* c.4539 + 2028C > T, based on age at disease onset, 4 patients who carry this variant *in trans* with a null or null-like variant were compared with 12 previously reported double null/null-like individuals^15^, 28 individuals who carry mild variants *in trans* with a null or null-like variant (8 from the Target 5000 cohort, 20 previously reported^15^) and 29 individuals who carry intermediate variants *in trans* with a null or null-like variant (5 from the Target 5000 cohort, 24 previously reported^15^) (Fig. 6). Median ages of onset were compared across all groups using a Kruskal–Wallis ranked sum test (p = 0.0000008837), with post hoc analysis carried out using a pairwise Wilcoxon rank sum test, correcting for multiple testing using the Holm-Bonferroni method. Only one individual from each pedigree was included in the tests in order to control for factors such as shared environment, which may influence phenotypic outcome. A significant difference was observed between the median age at onset of those who carried *ABCA4* c.4539 + 2028C > T *in trans* with a null or null-like variant and those who were carried two null/null-like variants (p = 0.0082). The median age at onset of those who carried *ABCA4* c.4539 + 2028C > T *in trans* with a null or null-like variant was 14 (range = 12–17, n = 4), while the median age at onset of those who carried two null/null-like variants was 6 (range = 4–10, n = 12). Those who carried a variant of intermediate severity *in trans* with a null or null-like variant had a median age of onset of 16 (range = 6–53, n = 28). The median age at onset for those who carried a mild variant *in trans* with a null or null-like variant was 25 (range = 11–56, n = 28). A significant difference was not observed between the median age at onset of those who carried *ABCA4* c.4539 + 2028C > T *in trans* with a null or null-like variant and those who carried a mild or intermediate variant *in trans* with a null or null-like variant (p = 0.1042 and p = 0.6581 respectively) (Fig. 6). Comparison of a variant of interest found *in trans* with a null or null-like allele with individuals who carry two null/null-like variants has been used to interrogate variant severity previously^15,36^. However, as age at onset is subject to recall bias (the proband may not have attended the current clinical centre at first presentation) it cannot be used in isolation as a definitive marker of variant severity.

The ages at onset of two patients homozygous for *ABCA4* c.4539 + 2028C > T were then compared to the four individuals who carried this variant *in trans* with a null or null-like variant. The homozygotes had a median age at onset of 22.5 (19 and 26) versus a median age at onset of 14 (range = 12–17, n = 4) in those who carry *ABCA4* c.4539 + 2028C > T *in trans* with a null or null-like variant.

### Variant assessment in accordance with the American College of medical genetics and genomics guidelines

The publication of the ACMG guidelines on variant interpretation has helped steer the assessment of potentially pathogenic variants in the medical genetics community since 2015^46^. Here we present several lines of evidence based on these principles that have been implemented to interpret the pathogenicity of the *ABCA4* c.4539 + 2028C > T variant. (1) PS3: Well-established in vitro or in vivo functional studies supportive of a damaging effect on the gene or gene product^25^. (2) PS4 (moderate): The prevalence of the variant in affected individuals is significantly increased compared to the prevalence in controls. However, as this is a very rare variant, this criterion is being used as moderate evidence based on observation of the variant in multiple unrelated patients with the same phenotype and almost complete absence in controls (1/816 Irish control alleles). (3) PM2: This variant is found at an extremely low frequency in the gnomAD database^42^ (0.00006371). (4) PM3: STGD1 is a recessive disorder, and this variant has been detected *in trans* with a pathogenic *ABCA4* variant multiple times. Therefore, this variant can be designated a pathogenic status based on the combination of one strong and 3 moderate lines of evidence.

## Discussion

In this study, we collated data on Irish participants with STGD1 who possess a pathogenic deep-intronic *ABCA4* variant, c.4539 + 2028C > T representing the largest cohort of such patients identified globally to date. We clearly illustrate the enrichment of *ABCA4* c.4539 + 2028C > T in the Irish STGD1 population, through analysis of this group and examination of the literature and databases for additional cases globally. A detailed genotype–phenotype analysis was performed suggesting that this deep intronic *ABCA4* variant results in a phenotype between mild and intermediate severity. Homozygotes present during adulthood and display well-circumscribed, localized, profound macular atrophy with minimal fleck lesions.

*ABCA4* c.4539 + 2028C > T was first described by Braun et al.^33^ in a study that described some of the first deep-intronic variants in *ABCA4.* Functional analysis was performed by Albert et al.^25^. RNA was first extracted from patient fibroblast cell lines, but aberrantly spliced product was not detected. To further investigate retina-specific splicing defects, RNA extracted from patient derived PPCs was examined and a 345 nucleotide pseudoexon inclusion was observed in ~ 7.5% of all transcripts and ~ 15% of transcripts derived from the *ABCA4* c.4539 + 2028C > T allele. This phenomenon, wherein a model that better recapitulates the patient displays a splice defect where one was not previously detected, has been noted previously^25,47^. The presence of *ABCA4* c.4539 + 2028C > T is predicted to create one SC35 and two SRp40 motifs by ESE finder 3.0^48^. *ABCA4* c.4539 + 2028C > T and *ABCA4* c.4539 + 2001G > A were the first reports of a pseudoexon inclusion in inherited retinal disease due to the creation of novel exon splice enhancer motifs, as opposed to the direct creation of cryptic splice acceptor/donor sites^25^. The neighboring c.4539 + 2001G > A variant that also results in the 345-nt pseudo-exon insertion showed 75% wild-type transcript though genotype–phenotype correlations classified it as ‘severe’. Given the nature of c.4539 + 2028C > T and additionally its enrichment in the Irish STGD1 patient cohort, it was of interest to explore the phenotypic consequences of this variant in patients.

All patients harbouring *ABCA4* c.4539 + 2028C > T and a known disease-causing variant presented with phenotypes that fall on the spectrum of STGD1. No other pathogenic candidate variants were detected using a variety of sequencing procedures, such as target capture next-generation sequencing^49–51^, entire *ABCA4* gene sequencing^24^, and whole genome sequencing. All individuals exhibited FAF abnormalities, with this abnormality extending beyond the vascular arcades in 13/25 individuals. In general, a normal/mild peripheral phenotype was observed. However, severe atrophy of the central macula was detected in most cases. Four participants harboured *ABCA4* c.4539 + 2028C > T *in trans* with a null or null-like variant and two participants were found to carry the variant in a homozygous manner. These individuals were phenotypically compared with those harbouring mild or intermediate variants *in trans* with null or null-like variants. Age at onset, ERG amplitudes as well as FAF and OCT patterns were used to assess variant severity, suggesting that the phenotype of the individuals who were homozygous for *ABCA4* c.4539 + 2028C > T was most similar to those that had been described as intermediate previously^15^. Other lines of evidence support this hypothesis, for example, *ABCA4* c.4539 + 2028C > T was not detected *in trans* with a variant previously categorized as mild. This may be because when *in trans* with a mild variant, the level of functional ABCA4 protein produced is sufficient to prevent significant loss of vision and thus individuals did not present for examination. As mentioned previously, functional analyses in PPCs indicated that residual wild-type transcript is produced in the presence of *ABCA4* c.4539 + 2028C > T^25^. The resulting phenotype also greatly depends on the pathogenic variant found *in trans,* providing additional evidence of between mild and intermediate severity. This is supported by the phenotype of both homozygous individuals who were older at disease onset, have a more well-preserved peripheral phenotype, better ERG amplitudes (ERG group 1: normal ffERG) and better visual acuity over time than those who carry the variant *in trans* with a null or null-like variant. It is of note that the disease observed in the two homozygotes is not widespread within the retina, but the fovea and perifoveal area is profoundly impacted in both.

Population-specific studies have provided a wealth of data describing the genomic architecture of IRDs in particular countries, identifying variants present at distinctly higher frequencies than other cohorts^52–57^. Similarly, it has become apparent that *ABCA4* c.4539 + 2028C > T is enriched in the Irish STGD1 population. A total of 27 incidents of this variant have been identified in our cohort to date, by comparison with 15 incidents of the variant identified across 5 studies in 3,940 alleles tested globally^24,30,33,40,41^. The Irish cohort includes, to the best of our knowledge, the only two patients homozygous for this variant identified to date. A complex allele containing *ABCA4* c.4539 + 2028C > T and *ABCA4* c.302 + 68C > T, is frequently observed, but not in all incidents. Among the 27 *ABCA4* c.4539 + 2028C > T alleles detected in Irish STGD1 patients, 24 incidents of c.302 + 68C > T were also detected. The segregation of these two variants in a complex allele has been reported previously^30,33,44^ and also accounts for the majority of Irish cases with c.4539 + 2028C > T. In our cohort, although *ABCA4* c.4539 + 2028C > T could be observed both in the presence of *ABCA4* c.302 + 68C > T or in isolation, *ABCA4* c.302 + 68C > T was only observed as part of the complex allele. Importantly, for those with sequencing datasets that are limited to the exonic regions of *ABCA4,* c.302 + 68C > T may be captured due to the nature of target capture sequencing by hybridization and its proximity to exon 3. As *ABCA4* c.302 + 68C > T has not been shown to have a qualitative effect on the resulting mRNA^25^, directly sequencing for *ABCA4* c.4539 + 2028C > T, in cases where c.302 + 68C > T has been detected may aid in providing a more conclusive genetic diagnosis where one has not yet been determined. This approach adds value to existing exome or target panel data and may reduce the need for whole gene or whole genome sequencing to resolve partial STGD1 diagnoses, particularly in cases where the patient is of Irish descent. This is internationally relevant as approximately 10% of the population in some western countries, including the UK, US, Canada and Australia claim Irish heritage^58–60^. Pedigrees harbouring *ABCA4* c.4539 + 2028C > T account for 14% of resolved STGD1 pedigrees in the Irish cohort to date.

The identification of IRD patients homozygous for putative pathogenic variants is imperative, as these Mendelian conditions present with phenotypes that can be greatly attributed to that particular variant, as illustrated by other STGD1 studies^15,36^. This is particularly true of splice altering variants. Given their mostly intronic nature, pathogenicity can currently only be proven by in vitro and ex vivo models that recapitulate the patient context to a limited extent, as demonstrated by the lack of identifiable splice defect in patient derived fibroblasts harbouring *ABCA4* c.4539 + 2028C > T and low percentage of mutant transcript in PPCs^25^. The participation of both homozygous patients in this study provides unique and invaluable insights into the phenotypic outcome of this variant, facilitating true variant categorisation and interpretation. In addition, as increasing numbers of homozygous patients are identified and if their phenotypes vary despite non-detection of other pathogenic variants, they may provide unique insights into potential modifier variants, *ABCA4* c.4539 + 2028C > T appears to result in a small area of profound central macular atrophy without evidence of disease in the peripheral retina. Describing this genotype–phenotype correlation may enable more accurate clinical phenotyping, facilitating greater positive pre-test probability for diagnosis of this particular allele, particularly in unresolved mono-allelic cases where Irish ancestry is noted. However, as this study examines the effect of a rare variant on a rare disease in a small number of individuals, it must be noted that caution should be taken when attributing severity to individual alleles. There is variance between the individuals in each group and the numbers of individuals in each group. While cumulatively these patient groups represent by far the largest patient cohort globally for the *ABCA4* c.4539 + 2028C > T variant, the numbers are still limited as expected for a rare disease. However, the study provides insights into the disease features associated with the *ABCA4* c.4539 + 2028C > T variant. In addition, interrogation of this variant in ex vivo retinal organoids, three dimensional structures derived from patient pluripotent stem cells, may more accurately illustrate the level of mutant versus wild-type transcript produced. This may more accurately recapitulate the splice defect observed in the retina of patients. Moreover, while extremely heritable, inherited retinal diseases may not be strictly Mendelian at times. Modifier variants, environmental influences and other factors are likely to affect phenotypic outcome, with a recent study indicating that sex may play a modifying role in STGD1^17,19^*.*

A variety of therapies are under consideration for STGD1. Preclinical trials have employed lentiviral vectors, chosen based on their cargo capacity relative to the size of the *ABCA4* gene, resulting in reduced levels of lipofuscin^61^. A phase I/II clinical trial of SAR422459 is investigating the safety and efficacy of subretinal injection and lentiviral-mediated delivery of *ABCA4*^62^*.* Additionally, dual adeno-associated virus (AAV) therapies for STGD1 are being considered^63,64^. Interestingly, the possibility of subretinal transplantation of human embryonic stem cell-derived retinal pigment epithelium cells (MA09-hRPE) is also under investigation for cases with more advanced macular/foveal atrophy. A phase I/II clinical trial has resulted in no adverse immune effects in 7/7 patients and 3 of these 7 reported increased visual acuity during a 12-month trial^65,66^. In addition, intronic variants such c.4539 + 2028C > T represent ideal candidates for antisense oligonucleotide (AON)-based therapy, employing short synthetic RNA molecules to restore correct splicing patterns. Promising pre-clinical results modulating the splice defect associated with *ABCA4* c.4539 + 2028C > T have been obtained, rescuing 80% of the observed splice defect^25^. Extensive work has been carried out on the modulation of splice defects that cause other forms of IRD, namely on the *USH2A*^67^*, CHM*^68^ and *OPA1*^69^ genes. Studies are being carried out on a recurring deep-intronic variant (c.2991 + 1655A > G) in *CEP290* that causes Leber congenital amaurosis^47,70–74^. Like *ABCA4* c.4539 + 2028C > T, this variant leads to pseudoexon incorporation in the mature mRNA transcript, and retinal-like cells (retinal organoids) showed a stronger pseudo-exon insertion then non-retinal cells^47^. AONs designed to correct this splice defect have displayed restoration of wild-type mRNA both in vitro and *in vivo*^75^. Furthermore, this therapeutic strategy has produced promising results in the first clinical trial using AONs for IRDs, with a phase II trial now underway^76^.

In this study, we provide detailed genetic and clinical characterization of 25 individuals with STGD1 in Ireland who carry *ABCA4* c.4539 + 2028C > T, which we define as a having a macula-centred phenotype of between mild and intermediate severity. We also illustrate the enrichment of this variant in Ireland and describe a specific clinical phenotype in homozygotes as a result of this variant. Notably these findings represent important considerations for clinicians when making an accurate phenotypic diagnosis, as well as for geneticists in ascertaining a genetic diagnosis in unresolved STGD1 cases, particularly in those of Irish descent. This study also underscores the necessity of intronic as well as exonic sequencing in previously unresolved STGD1 cases. Given the widespread availability of DNA sequencing in this era of genomic medicine, it is imperative that patients’ interests are at the forefront, with access to novel therapies dependent on a genetic diagnosis. With this wealth of genetic information comes the responsibility to try to establish accurate associations between genotypes and phenotypes. The current study aids this important objective with respect to STGD1. The findings should serve to facilitate additional harmonized clinical and genetic diagnoses as well as improving both clinician and patient understanding of disease progression and management in STGD1 patients who carry *ABCA4* c.4539 + 2028C > T.

## Materials and methods

### Participants

Participants were recruited through The Royal Victoria Eye and Ear Hospital and The Mater Misericordiae University Hospital, Dublin as part of the Target 5000 study^77^. The participant cohort consisted of 25 individuals across 18 pedigrees clinically diagnosed with STGD and found to possess at least one allele harbouring the *ABCA4* c.4539 + 2028C > T variant. 13 unaffected relatives were also recruited in order to determine the phase of variants identified. In addition, 15 affected participants carrying two *ABCA4* variants of previously categorised severity and null/null-like variants^15^ were recruited from Target 5000 and their phenotypic data used for comparative purposes to determine the severity the *ABCA4* c.4539 + 2028C > T variant. Previously published data on age at onset from 56 affected individuals carrying variants of known severity was also included in this study. Of the 25 participants with *ABCA4* c.4539 + 2028C > T, 13 were female while 12 were male, with a median age at last examination of 38 years (range: 13–70).

### Clinical assessment

All study participants had a clinical diagnosis of either STGD1 or cone-rod dystrophy. Informed consent was obtained from each participant and the appropriate assessments were performed in order to determine the patient’s phenotype. Clinical data collected and analysed included sex, age at last examination, age at onset of disease, visual acuity (Revised 2000 early treatment diabetic retinopathy study (ETDRS) charts, Precision Vision, La Salle, IL, USA), visual fields (Goldmann perimeter 940 (iv4e, i4e and 04e targets), Haag-Streit AG, Köniz, Switzerland), colour vision (Lanthony desaturated D-15 panel under standardised lighting conditions, Gulden Ophthalmics, Elkins Park, PA, USA), dilated slit lamp biomicroscopy, colour fundus photography and fundus autofluorescence (FAF) (Topcon CRC50DX/Optos Daytona, Topcon Great Britain Ltd., Berkshire, England/Optos plc, Dunfermline, Scotland), optical coherence tomography (OCT) (Cirrus 5000, Carl Zeiss Meditec, Berlin, Germany), and electroretinography (ERG) Roland Consult RETI-port retiscan, Brandenburg an der Havel, Germany), where available. The least affected reading was used for data illustration purposes in all cases.

### Sequencing procedures

13 affected individuals with *ABCA4* c.4539 + 2028C > T underwent target capture next generation sequencing of the exons and known pathogenic intronic regions of *ABCA4* as described previously^49–51^, 9 affected individuals with *ABCA4* c.4539 + 2028C > T underwent whole-gene single molecule molecular inversion probe (smMIP) based sequencing of *ABCA4* as well as 40 kb of flanking sequence as described previously^24^, including both homozygotes. Two individuals with *ABCA4* c.4539 + 2028C > T underwent direct Sanger sequencing. (Eurofins Genomics, Germany). The remaining individual with c.4539 + 2028C > T underwent whole genome sequencing (WGS)^78^. WGS was performed by BGI (Hong Kong, China) on a BGISeq500 using 2 × 100 bp paired end reads, with a 30-fold minimal median coverage per genome. Burrows-Wheeler Aligner was utilized to map data to the human genome (GRCh37). The quality of the WGS data was based on insert size, percentage mapped reads, percentage duplicated mapped reads, coverage, bases with > 20X coverage and error rate, which were evaluated using Qualimap V.2.2.1^79^. Variant calling was performed by xAtlas V.0.1 and annotated in-house with data such as variant type, in silico pathogenicity prediction scores and population frequency. Variant validation was carried out by amplifying the surrounding region via polymerase chain reaction (PCR). This was followed by Sanger sequencing (Eurofins Genomics, Germany). Primers for use in this procedure were manufactured by Sigma Aldrich (Gillingham, England, UK). New England Biolabs Inc.’s (Ipswich, MA, USA) Q5 High-Fidelity 2 × Master Mix was used to amplify the regions surrounding the variant. Where available, relatives samples were processed similarly using either the Phusion Human Specimen Direct PCR Kit or the Phusion Blood Direct PCR Kit (Thermo Scientific, MA, USA) where appropriate. The relevant accession numbers for *ABCA4* in this study are NM_000350.2 and NC_000001.11.

### Variant categorisation

Variants designated “mild”, “intermediate” or “null-like” were based on a previous comprehensive study by Fakin et al.^15^. Variants categorised as intermediate: *ABCA4* c.6079C > T,p.(Leu2027Phe), c.4139C > T,p.(Pro1380Leu), c.3113C > T,p.(Ala1038Val) and c.3322C > T,p.(Arg1108Cys). Variants categorised as mild: *ABCA4* c.5882G > A,p.(Gly1961Glu) and c.5603A > T,p.(Asn1868Ile). Variants categorised as null or null-like: c.6449G > A,(p.Cys2150Tyr), c.2564G > A,p.(Trp855*), c.4469G > A,p.(Cys1490Tyr), c.1906C > T,(p.Gln636*), c.5312 + 1G > A,p.(?), c.2160 + 1G > C,p.(?), c.4577delC,p.(Thr1526Argfs*10), c.1222C > T,(p.Arg408*), c.4234C > T,(p.Gln1412*), c.4320delT,p.(Phe1440Leufs*6), c.2041C > T,p.(Arg681*), c.735 T > G,p.(Tyr245*), and c.3329-1G > A,p.(?) Variant categorisation of c.4539 + 2028C > T was carried out by comparison of participants homozygous for c.4539 + 2028C > T to participants harbouring c.4539 + 2028C > T *in trans* with a null or null-like variant to determine the effect of the variant in isolation. In addition, participants harbouring c.4539 + 2028C > T *in trans* with a null or null-like variant were phenotypically compared to those who carry intermediate or mild variants *in trans* with a null or null-like variant to gauge severity.

### Statistical analyses and data visualisation

Statistical analyses and data visualisation were performed using R version 4.0.3 (R Core Team (2020). R: A language and environment for statistical computing. R Foundation for Statistical Computing, Vienna, Austria. URL https://www.R-project.org/). The median age at onset of those harbouring *ABCA4* c.4539 + 2028C > T were compared with the age at onset of individuals who carried two null/null-like variants, those who carried mild variants *in trans* with a null or null-like variants and those who carried intermediate variants *in trans* with a null or null-like variants previously reported^15^ using a Kruskal–Wallis ranked sum test with post hoc analysis carried out using a pairwise Wilcoxon rank sum test, correcting for multiple testing using the Holm-Bonferroni method. Only one affected individual from each pedigree was included in these analyses in order to exclude other factors that may influence the phenotypic outcome, such as shared environment.

### Editorial policies and ethical considerations

Prior to commencement, The Research and Medical Ethics committee of the Royal Victoria Eye and Ear Hospital (13-06-2011: HRA-POR201097) and the Institutional Review Board of the Mater Misericordiae University Hospital and Mater Private Hospital (MMUH IRB 1/378/1358), Dublin, Ireland, awarded ethical approval for this study. Written informed consent was obtained in accordance with the Declaration of Helsinki. All participants gave written informed consent before commencement of the study.

## Supplementary Information

Supplementary Information.

## Data Availability

All data relevant to the study are included in the article and supplementary data. The datasets generated and/or analysed during the current study are available in the Leiden Open Variation Database (LOVD) (https://grenada.lumc.nl/LSDB_list/lsdbs/ABCA4). The sequencing data is not publicly available as this could compromise research participant privacy. Sequencing data may become available upon a data transfer agreement approved by local ethical committees. Patient sample identifiers from this study can be released upon reasonable request to the corresponding local DNA identifier. Other data requests can be addressed to the corresponding author (L.W.) and will be addressed upon reasonable request.
