# Supplementary material for: Detailed analysis of an enriched deep intronic ABCA4 variant in Irish Stargardt disease patients
Source: Sci Rep. 2023 Jun 9;13:9380. doi: 10.1038/s41598-023-35889-9 (PMC10256698; doi:10.1038/s41598-023-35889-9)
Supplement: Supplementary file 1 — Supplementary Information. [file 41598_2023_35889_MOESM1_ESM.docx]

Enrichment of *ABCA4* c.4539+2028C>T in Stargardt disease patients in Ireland - a detailed genotype-phenotype analysis.

**Supplemental Materials.**

**Table S1.** PCR primers used to amplify regions of *ABCA4* harboring pathogenic variants.

| **Amplicon Region** | **Forward** | **Reverse** | **Variant(s)** |
| --- | --- | --- | --- |
| ***ABCA4* Intron 30** | GGGACCAAGGACCAACACTA | GCTCATCTGCTGCATCATAGG | **c.4538+2028C>T** |
| ***ABCA4* Intron 3** | AAGGCAGTGGACACATCTCT | CTCCAGGGGATCTTCTGCAA | **c.302+68C>T** |
| ***ABCA4* Exon 35** | TGGTATGTGAAGTGTGAGGCA | ACCAAACCCTTCCTGCTTCT | **c.4918C>T** |
| ***ABCA4* Exon 3** | TAAGAGGTTAGGGGCTCAGC | GAGCTTGAGAGAAGAGGCCA | **c.161G>A**  **c.223T>G** |
| ***ABCA4* Exon 22** | GGGAAGTAGGTTGCATCATCAG | GCCTTTCTCTTCCTCACCCT | **c.3322C>T  c.3212C>A** |
| ***ABCA4* Exon 40** | CCTGAGGAAAGAAATGACCATGT | CTGGAGAGGATTAGGGTGCC | **c.5603A>T** |
| ***ABCA4* Exon 47** | TGGCCTTCTCCATCTGCTG | ACATCCCACAGGCAAGAGAT | **c.6472A>G**  **c.6449G>A** |
| ***ABCA4* Exon 28** | AGTGAAGTGGGAAGGTCAGG | AGATTCTTCAGTGGCCACCA | **c.4222T>C**  **c.4139C>T** |
| ***ABCA4* Exon 14** | GCTAGATGTCACGCTCTCCT | TGATCATCCTGAACCGCTGT | **c.2160+1G>C** |
| ***ABCA4* Exon 16** | GGCTGGGGATCTGAAGAACT | CCCTCCCCTCTACCTCGAG | **c.2453G>A** |
| ***ABCA4* Exon 21** | GCAGGGAAAATGATCTGGGG | TGTATCATAAACACCACCACAGT | **c.3113C>T** |

**Table S2.** Variants identified in the Irish genetically screened STGD1 cohort (n=214) and their percentage frequency within this population. The relevant accession number is NM_000350.2.

| Variant | % Frequency |
| --- | --- |
| c.5603A>T | 13.29 |
| c.161G>A | 6.74 |
| c.4139C>T | 5.20 |
| c.4539+2028C>T | 4.82 |
| c.4577C>T | 4.05 |
| c.302+68C>T | 3.85 |
| c.2588G>C | 3.85 |
| c.3322C>T | 3.08 |
| c.5882G>A | 2.89 |
| c.4222T>C | 2.70 |
| c.3056C>T | 2.70 |
| c.4918C>T | 2.70 |
| c.6079C>T | 2.31 |
| c.5461-10T>C | 2.31 |
| c.3323G>A | 1.54 |
| c.3113C>T | 1.54 |
| c.4253+43G>A | 1.35 |
| c.4363T>C | 1.35 |
| c.4469G>A | 1.35 |
| c.2827C>T | 1.16 |
| c.1317G>A | 0.96 |
| c.5196+1137G>A | 0.96 |
| c.6098T>G | 0.96 |
| c.5917delG | 0.77 |
| c.1622T>C | 0.77 |
| c.1715G>A | 0.77 |
| c.6449G>A | 0.77 |
| c.5714+5G>A | 0.77 |
| c.5929G>A | 0.77 |
| c.1519G>T | 0.58 |
| c.1253T>C | 0.58 |
| c.4685T>C | 0.58 |
| c.1906C>T | 0.58 |
| c.4957G>A | 0.58 |
| c.1804C>T | 0.58 |
| c.6089G>A | 0.58 |
| c.1018T>C | 0.39 |
| c.2041C>T | 0.39 |
| c.5113C>T | 0.39 |
| c.466A>G | 0.39 |
| c.735T>G | 0.39 |
| c.4861A>T | 0.39 |
| c.223T>G | 0.39 |
| c.2894A>G | 0.39 |
| c.5351T>C | 0.39 |
| c.1222C>T | 0.39 |
| c.5921A>G | 0.39 |
| c.6472A>G | 0.39 |
| c.4225A>G | 0.39 |
| c.3329-1G>A | 0.39 |
| c.5908C>T | 0.39 |
| c.5312+3A>T | 0.39 |
| c.4254-5T>A | 0.39 |
| c.3481C>A | 0.19 |
| c.2564G>A | 0.19 |
| c.5312+1G>A | 0.19 |
| c.1532G>A | 0.19 |
| c.3292C>T | 0.19 |
| c.4577del | 0.19 |
| c.868C>T | 0.19 |
| c.752delT | 0.19 |
| c.4234C>T | 0.19 |
| c.2549A>G | 0.19 |
| c.191C>T | 0.19 |
| c.731T>C | 0.19 |
| c.4793C>A | 0.19 |
| c.6658C>T | 0.19 |
| c.3754G>T | 0.19 |
| c.4919G>A | 0.19 |
| c.5594dupA | 0.19 |
| c.1933G>A | 0.19 |
| c.2382+1dup | 0.19 |
| c.3137T>G | 0.19 |
| c.286A>G | 0.19 |
| c.3305A>T | 0.19 |
| c.2701A>G | 0.19 |
| c.4320del | 0.19 |
| c.3386G>T | 0.19 |
| c.1037A>C | 0.19 |
| c.5644A>G | 0.19 |
| c.3364G>A | 0.19 |
| c.5348T>C | 0.19 |
| c.768G>T | 0.19 |
| c.655A>T | 0.19 |
| c.1817G>A | 0.19 |
| c.286A>C | 0.19 |
| c.2852T>C | 0.19 |
| c.2242T>C | 0.19 |
| c.223T>C | 0.19 |
| c.6221G>A | 0.19 |
| c.2160+1G>C | 0.19 |
| c.5898+5G>A | 0.19 |
| c.2453G>A | 0.19 |
| c.550T>C | 0.19 |
| c.5917del | 0.19 |
| c.5773A>G | 0.19 |
| c.185C>T | 0.19 |
| c.4319C>T | 0.19 |
| c.2483C>T | 0.19 |
| c.6743T>C | 0.19 |
| c.869G>A | 0.19 |
| c.1805G>A | 0.19 |
| c.3212C>A | 0.19 |
| c.4468T>C | 0.19 |
| c.455G>A | 0.19 |
| c.5196+1G>A | 0.19 |
| c.5329A>G | 0.19 |
| c.1865G>A | 0.19 |
| c.3758C>T | 0.19 |
| c.2311A>T | 0.19 |
| c.5381C>A | 0.19 |
| c.6416G>C | 0.19 |
| c.551C>G | 0.19 |
| c.4326C>A | 0.19 |
| c.5693G>A | 0.19 |
| c.6286G>A | 0.19 |
| c.587C>T | 0.19 |
| c.6448T>C | 0.19 |
| c.3552C>G | 0.19 |
| c.4771G>A | 0.19 |
| c.4283C>T | 0.19 |


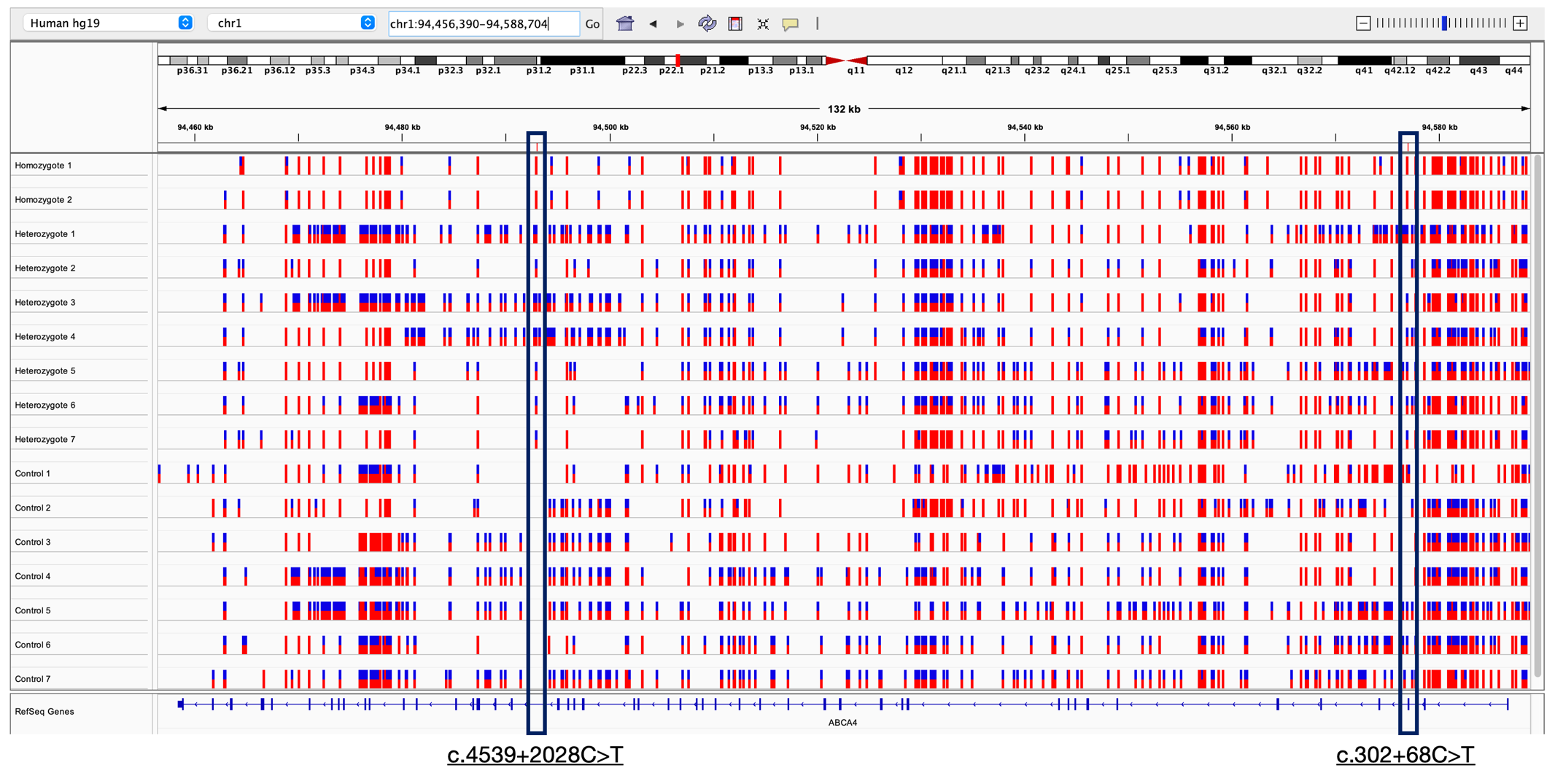


**Figure S1. Visualisation of variant call format (VCF) files from 2 affected individuals homozygous for c.4539+2028C>T, 7 affected individuals heterozygous for c.4539+2028C>T and 7 affected individuals who do not carry the variant (‘’controls’’).** This figure illustrates variation present across the *ABCA4* gene in these individuals. Areas highlighted in red alone represent regions where variation is present homozygously. Areas highlighted in red and blue represent regions where variation is present heterozygously. Areas in white represent regions where no variation is present. Regions harbouring c. 4539+2028C>T and c.302+68C>T are highlighted. It is clear from this image that the homozygous individuals are highly similar in terms of variation.
